# An Unusual Case of Maxillary Sinus Odontogenic Keratocyst: An Insightful Report With Review of the Literature

**DOI:** 10.7759/cureus.37357

**Published:** 2023-04-10

**Authors:** Shruti Singh, Priti Shukla, Ravinder S Bedi, Shruti Gupta, Shivesh Acharya

**Affiliations:** 1 Oral and Maxillofacial Pathology, All India Institute of Medical Sciences, Raebareli, IND; 2 Orthodontics and Dentofacial Orthopaedics, All India Institute of Medical Sciences, Raebareli, IND; 3 Oral and Maxillofacial Surgery, All India Institute of Medical Sciences, Raebareli, IND; 4 Pathology, All India Institute of Medical Sciences, Raebareli, IND; 5 Pediatric Dentistry, All India Institute of Medical Sciences, Raebareli, IND

**Keywords:** contrast-enhanced ct, pterygoid plate, carnoy’s solution, odontogenic keratocyst, maxillary sinus

## Abstract

Maxillary sinus odontogenic keratocyst (OKC) is very rare and occupies less than 1% of the total OKC cases reported in the literature. OKCs have characteristic features that are unique compared to other cysts of the maxillofacial region. Considering their peculiar behaviour, varied origin, debated development, discourse treatment modalities, and high recurrence rate, OKCs have been a subject of interest for various oral surgeons and pathologists globally. This case report presents an unusual case of invasive maxillary sinus OKC into the orbital floor, pterygoid plates, and hard palate in a 30-year-old female. The case report confers that cystic maxillary sinus lesions should always be treated very extensively irrespective of the nature of the lesion as the site makes it highly susceptible to secondary infection and recurrence. The case also establishes a set of imaging modalities and specific treatment approaches to be followed for maxillary sinus OKC based on the literature of all the previous cases reported.

## Introduction

Odontogenic keratocyst (OKC) is a distinctive form of developmental odontogenic cyst comprising 12% of the entire jaw cysts [1]. Considering their peculiar behaviour, varied origin, debated development, discourse treatment modalities, and high recurrence rate, OKCs have been a subject of interest for various oral surgeons and pathologists globally [2].

The most common site as per the occurrence rate of OKC is the mandible (73%) compared with 27% in the maxilla [3]. OKCs of the maxillary sinus are even rarer with less than 1% of cases reported in the literature [4].

This case report is of an unusual presentation of an aggressive left maxillary sinus OKC in a 30-year-old female. Lesions involving the sinus always pose a diagnostic challenge as the margins are difficult to identify and sinus pathology of odontogenic origin holds high chances of secondary infection [5]. A brief review of all the reported maxillary sinus OKCs of the past 20 years has also been compiled with this case for understanding their behaviour patterns in terms of clinical, radiological, and treatment outcomes considering its rare occurrence in this site.

## Case presentation

**Figure 1:**
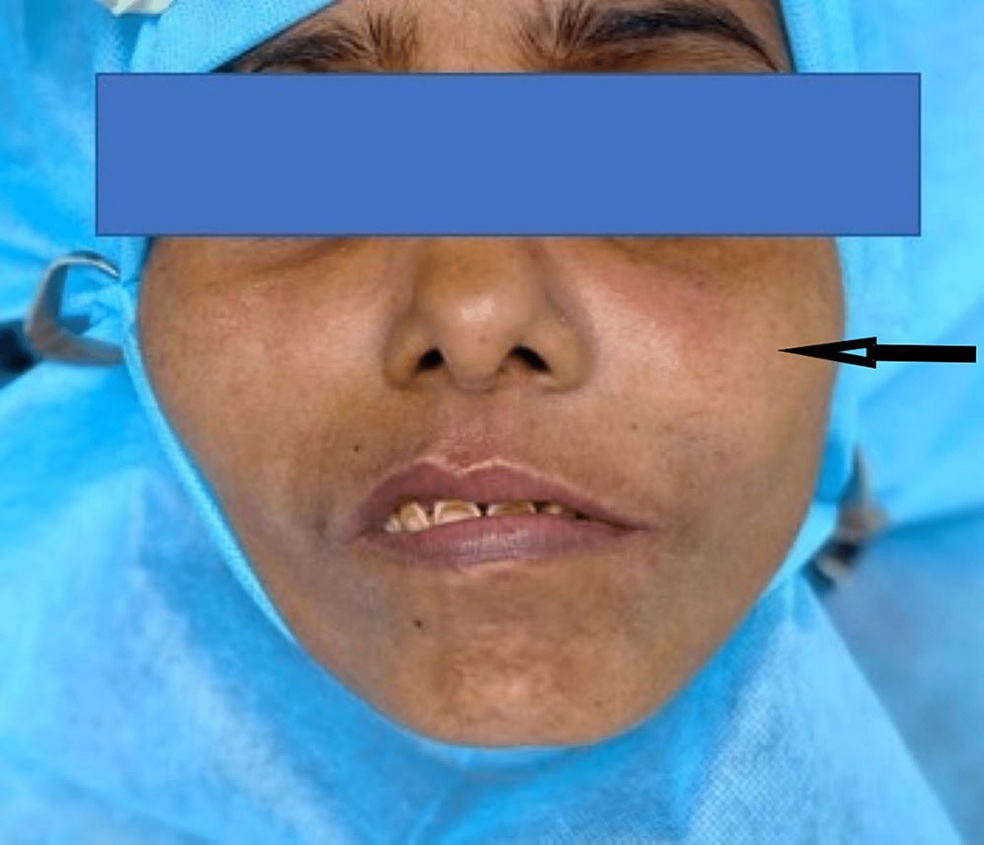
Extraoral clinical image Extraoral clinical image showing no visible gross facial asymmetry.

A 30-year-old female patient reported to the department of dentistry with the chief complaint of swelling on the left upper back region of the jaw for three years with mild heaviness for a month on the same side of the face. The patient gave a history of gradual increase in the size of the swelling to the present size with no association of pain or any other symptom. No significant personal, medical, or family history was reported. Slight facial asymmetry was present extraorally on the affected side (Figure 1).

**Figure 2:**
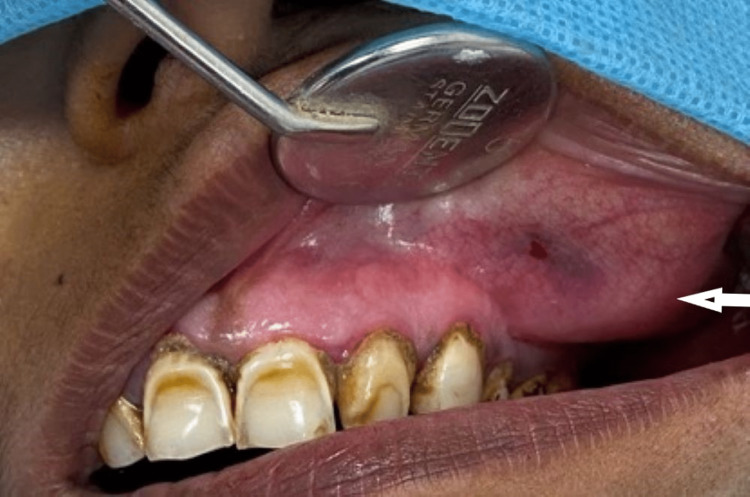
Diffuse swelling on the buccal aspect of the maxilla Diffuse swelling on the buccal aspect of the maxilla completely obliterating the vestibule region of 23-27.

Intraoral examination revealed diffuse swelling on the buccal aspect of the maxilla completely obliterating the vestibule region of 23-27 extending palatally involving the hard palate (Figure 2).

**Figure 3:**
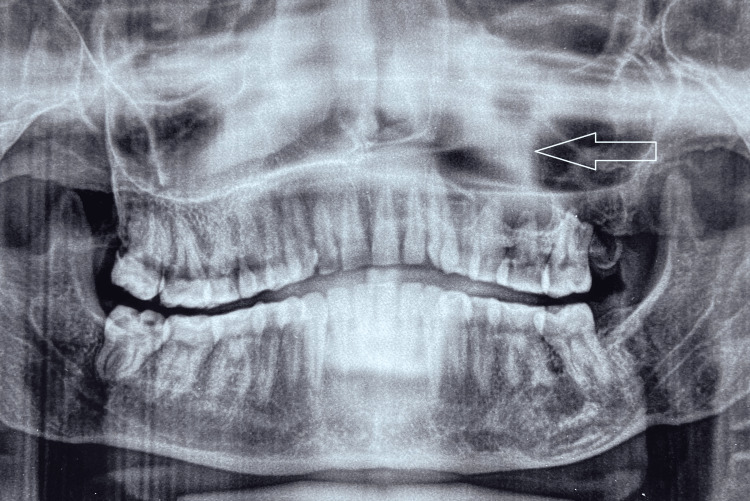
Pre-treatment orthopantomogram showing the expansion of the sinus lining

**Figure 4:**
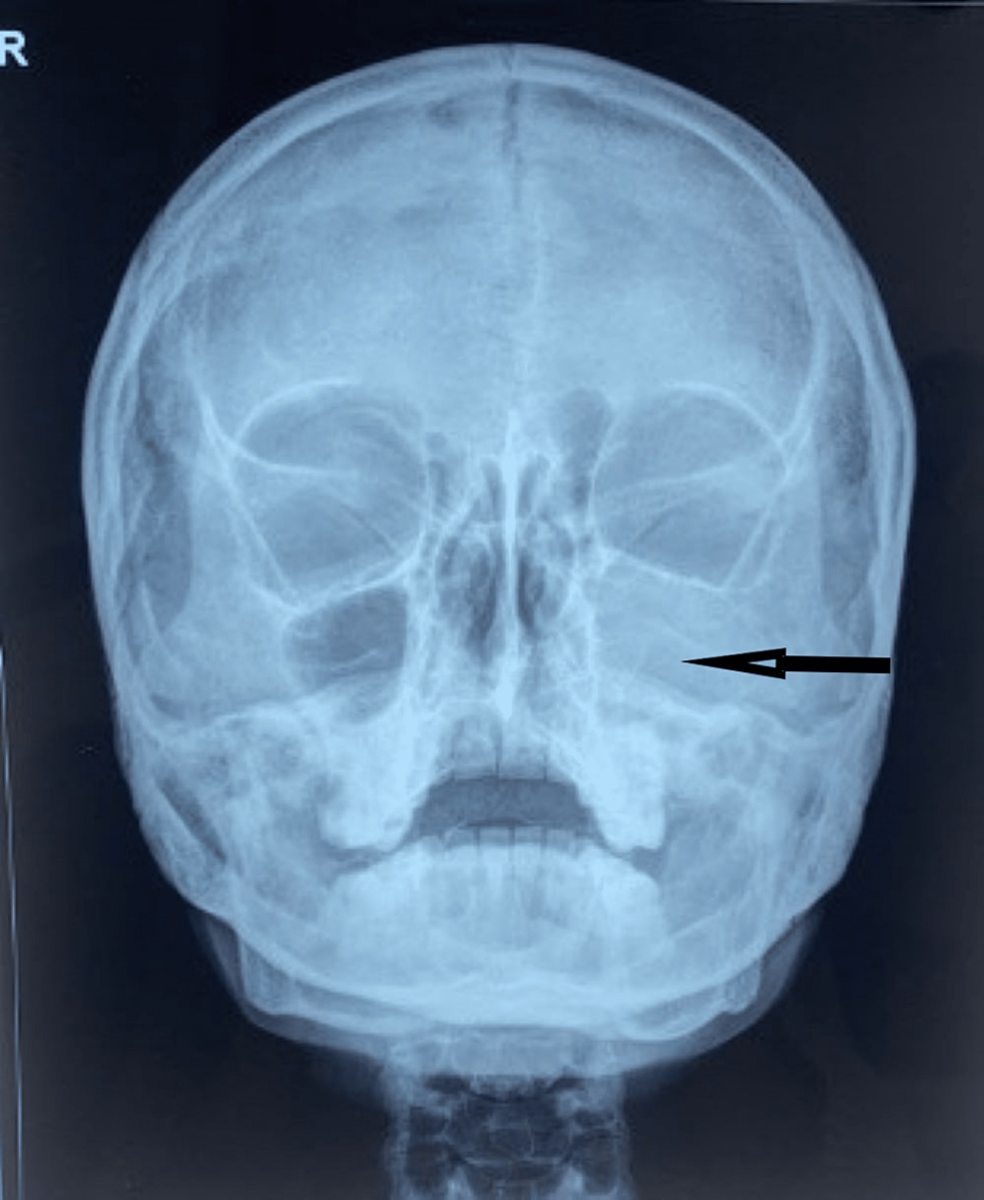
Paranasal sinus view showed complete obliteration of left maxillary sinus

On palpation, the swelling was non-tender and firm in consistency. Hard tissue examination revealed no clinical decay or mobility in the tooth in relation to the swelling, nor was there any history of discharge. The vitality of the teeth was assessed, and all the teeth were vital. Chronic generalized periodontitis with the presence of dental fluorosis was seen (Figure 2). Considering the site and extent of the swelling on intraoral examination without any oral symptoms, a clinical differential diagnosis of maxillary odontogenic sinusitis, periodontal cyst, and deep fungal infection of the maxillary sinus was considered. Hence, an orthopantomogram (OPG) (Figure 3) and a paranasal sinus view followed by contrast-enhanced computed tomography (CECT) were advised. The paranasal sinus view showed complete obliteration of the left maxillary sinus (Figure 4).

**Figure 5:**
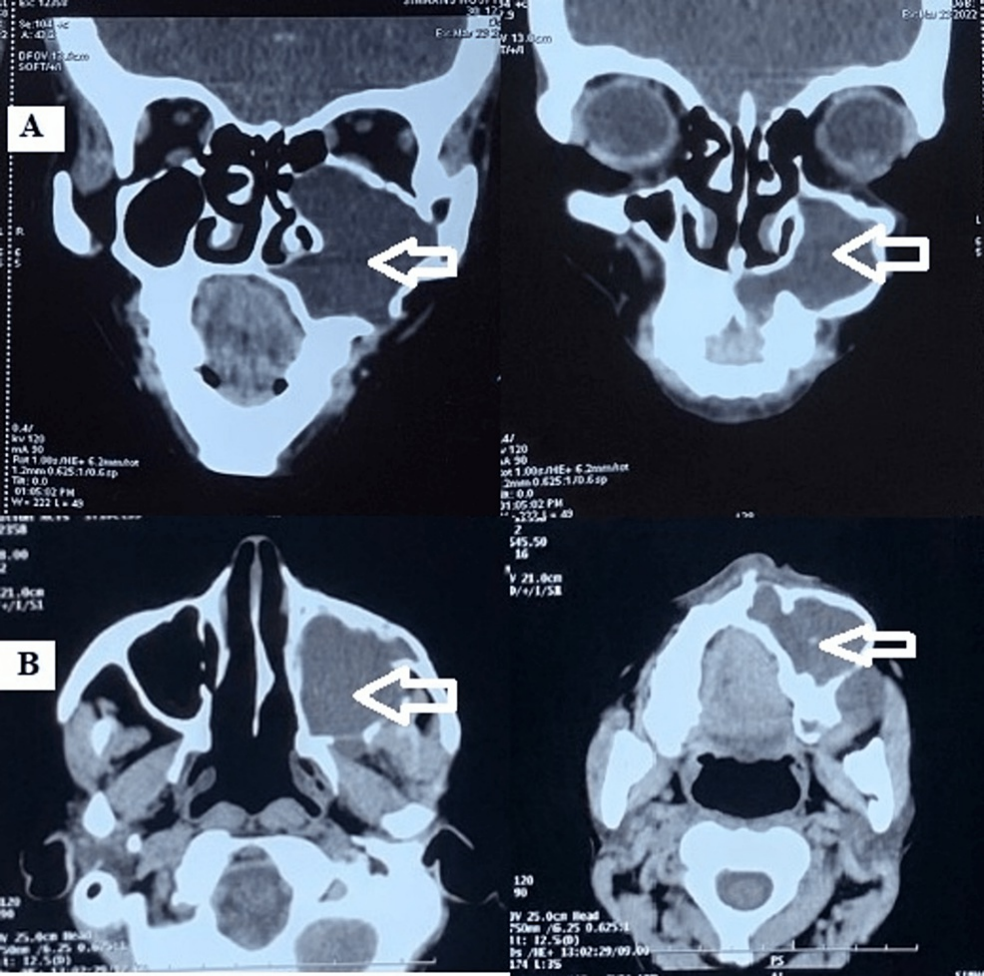
(A) Coronal and (B) axial CT: the expansile lytic lesion in the left alveolus with hypodense non-enhancing soft tissue component

CECT findings revealed an expansile lytic lesion in the left upper alveolus with cortical destruction. Hypodense non-enhancing soft tissue component was reported involving the roots of the teeth of the upper left posterior quadrant. The medial and lateral wall of the maxillary sinus was involved (Figure 5).

The lesion extended superiorly up to the orbit and medially involved the complete maxillary left sinus, inferiorly up to the hard palate and laterally up to the masseteric space (Figure 5A).

CT report concluded it to be a benign cystic lesion. Fine needle aspiration cytology (FNAC) was done, and aspirate yielded cream-coloured fluid and cytology revealed the presence of necrosis with cholesterol crystals.

**Figure 6:**
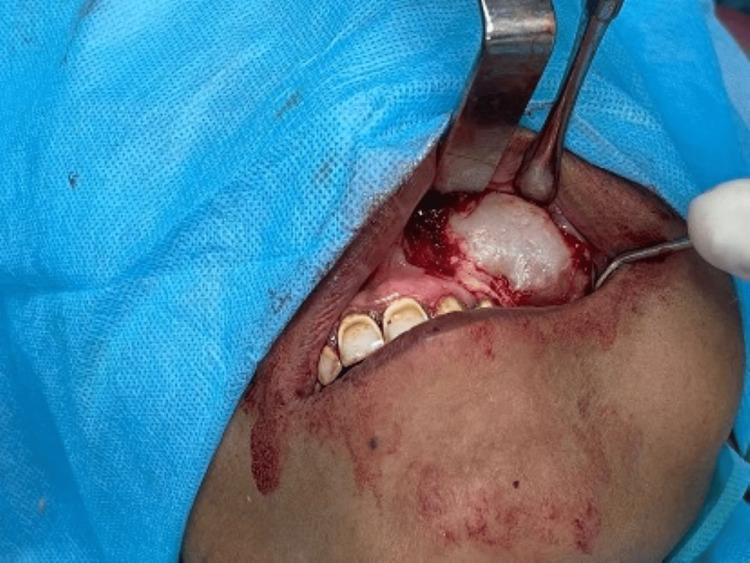
Surgical exploration through intra-oral approach: thinning and expansion of cortical plates

The surgical enucleation of the lesion was planned under local anaesthesia and antibiotic coverage with an intra-oral approach (Figure 6).

During surgical exploration, an abundance of white-cheesy content was collected. The lesion was highly aggressive with invasion into the pterygoid plates and orbital floor. The fragile cyst lining was enucleated, and curettage was done. Considering the invasive nature of the lesion, extended chemical cauterization was done using freshly prepared Carnoy’s solution. The cyst cavity was inspected to ensure complete excision, and an iodoform-medicated gauze pack was kept inside the cavity with one end out. This gauze pack was removed after three days.

**Figure 7:**
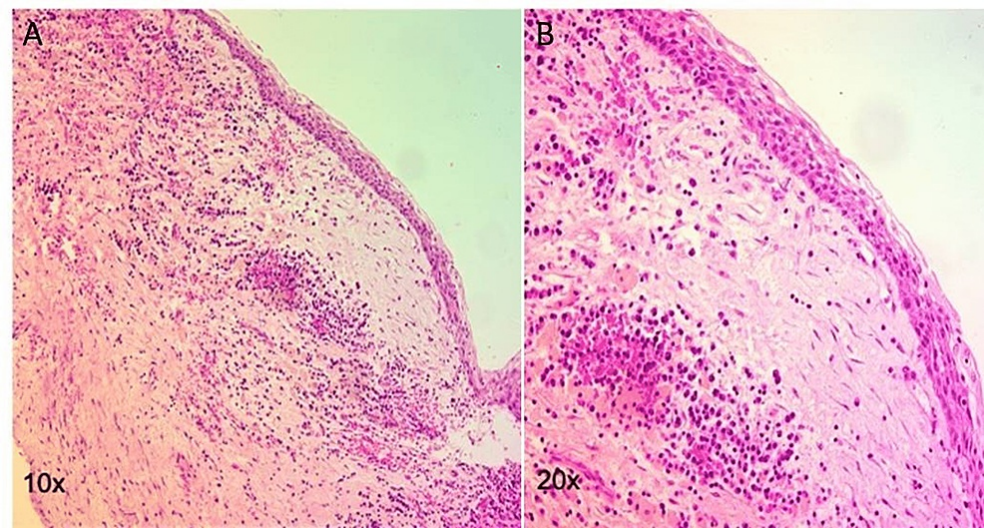
Fibro-dense cyst wall lined with para keratinization stratified squamous lining with palisaded cuboidal basal cells. The cystic wall is fibrous with focal areas of moderately dense chronic inflammatory cells

The enucleated specimen was sent for histopathological examination. Gross examination showed a thin cyst wall, which on microscopy revealed a cystic lining with parakeratinized stratified squamous epithelium with palisaded cuboidal basal cells. The cystic wall was fibrous with focal areas of dense chronic inflammatory infiltrate predominantly plasma cells and lymphocytes (Figure 7). Therefore, the histopathological diagnosis of OKC of the maxillary sinus was made.

**Figure 8:**
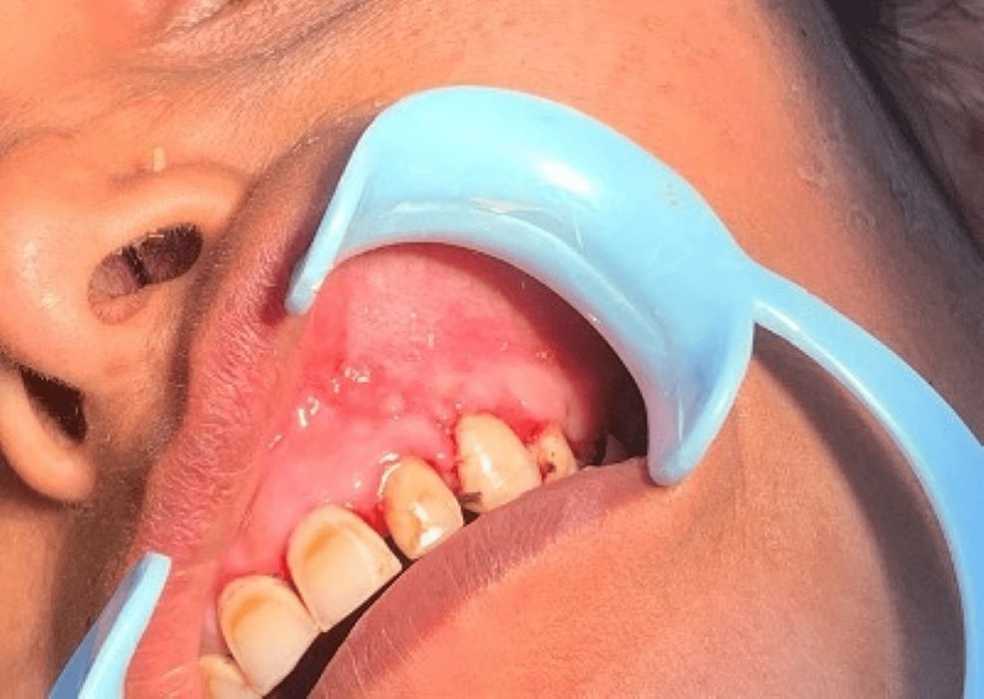
Post-operative follow-up image after four months post-surgery

The patient was followed up after four months and had no fresh complaints in accordance with the site of the lesion operated, and intraorally the site seemed to have restored its normal anatomy (Figure 8).

**Figure 9:**
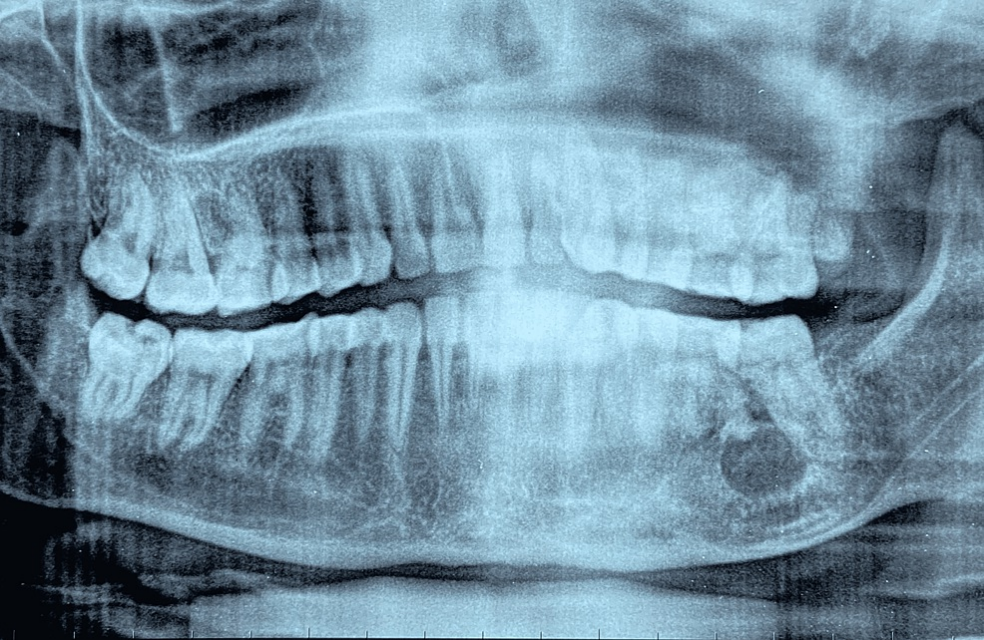
Post-operative orthopantomogram at six months follow-up showing no recurrence

The patient was kept in periodic follow-up, and OPG was repeated six months post-surgery, which showed no recurrence (Figure 9). CECT is to be repeated at the one-year post-surgery visit.

## Discussion

OKCs are the third most common developmental jaw cysts. The term was broadly used for all keratin-forming cysts during the 1950s. It was in 1956 that Phillipsen described OKC for epithelial developmental cysts [6]. OKCs show an occurrence of 65% to 83% in the mandible but their location in the maxillary site is conflicted [2,3]. OKCs that occur in regions other than the mandibular angle and especially those in the maxilla seem to be more related to systemic syndromes [3]. A literature search for case reports was conducted in PubMed, ClinicalKey, and Google Scholar databases using the keywords "maxillary sinus" and "invasive odontogenic keratocyst" and the available data for the last 20 years have been tabulated (Table 1).

**Table 1:** Tabulation of the literature on invasive maxillary sinus odontogenic keratocyst (OKC) case reports chronologically from 2001 to 2022

Year	Age/gender	Clinical features	Panoramic radiographic findings	Computed tomography findings	Treatment and recurrence
Silva et al. (2006) [7]	Case 1: 17 years/male; Case 2: 14 years/boy	Case 1 presented with symptoms of headache from a week with no swelling. Case 2 presented with intra-oral swelling with pus discharge.	Case 1: Lateral and post-anterior radiographs showed a discrete opaque mass with an image of the third molar in the left maxillary sinus. No CT was done.	Case 2: Ectopic second right upper molar involved by radiopacity filling in the upper posterior portion of the maxillary sinus.	Both were enucleated and curettage was done. Case 1: Follow-up of eight years with no recurrence. Case 2: Follow-up of five years with no recurrence.
Cakur et al. (2008) [4]	23 years/male	Pain and swelling in the right maxillary region accompanied by pus discharge.	Waters view of a lytic lesion in the right maxillary sinus with an impacted tooth.	Lytic lesion destroying and displacing the roof and lateral wall of the right maxillary sinus.	Complete excision through the Caldwell-Luc approach. No recurrence was reported after six months of follow-up.
Bhagavandas Rai et al. (2010) [2]	32 years/male	Diffuse painful extra oral swelling extending from left ala of the nose to left zygomatic prominence, intra-oral draining sinus in relation to maxillary left second premolar region.	Unilocular radiolucency extending from the root apex of the upper right canine to the mesial root of the upper left first molar. There was an inverted conical supernumerary tooth between the left central and lateral incisors.	No data available.	Surgical enucleation followed by chemical cauterization. Follow up for one year and no evidence of recurrence.
Okoje-Adesomoju et al. (2014) [8]	38 years/male	Diffuse bilateral maxillary swelling of three years involving the right infraorbital region resulting in epiphora. Growth of both swellings resulted in the mobility of teeth and pus discharge from 15.	No data available.	Complete destruction of the maxillae, palate, nasal septum, and nasal bone by a mass occupying the whole of the maxillae and maxillary antrum. Destruction of the floor of the orbit, ethmoid, sphenoid, and frontal sinuses as well as the roof of the frontal sinus.	Surgical decompression after shielding adjacent vital structures with Sofra-Tulle dressing and chemical cautery of the tumour bed was achieved with Carnoy’s solution. The cavities were then packed with argotone-soaked gauze to allow for proper haemostasis; this was left in place for three days. The patient was lost to follow-up.
Newaskar et al. (2016) [9]	21 years/male	Pain and extraoral swelling on the left middle third region of the face, intra-orally distal to the last tooth in the left upper back tooth region. The swelling was accompanied by pus discharge.	Haziness in both the right and left maxillary sinus and impacted teeth 18.	Hyperdense area obliterating complete left maxillary sinus causing lateral nasal wall at the level of the middle concha was destructed and showed displaced and impacted tooth 28.	Enucleation followed by peripheral ostectomy. No recurrence post two years of follow-up.
Maruthamuthu et al. (2017) [10]	45 years/female	Painless swelling on the right side of the face for the past six months with watering of the eyes for one month. History of surgery at the same site in 2005.	A well-defined multilocular radiolucent lesion with a sclerotic border present in the right maxilla extending between right 16 and 23 mesiodistally and superiorly involving the right orbit with discontinuity in the right infraorbital margin.	Two well-circumscribed lesions present in the anterior maxilla and the maxillary sinus region. Deviation of nasal septum towards the left side with obstruction of the right osteomeatal complex.	Both the lesions were enucleated with the maxillary sinus, were decompressed and then enucleated. It was a recurrent case but post-operative periodic follow was done.
Bastos et al. (2019) [11]	15 years/male	Maxillary swelling with no associated symptoms.	Tooth displaced to the orbital floor and presence of radiolucency in right maxillae.	Radiolucent and multilocular images with tooth displacement in proximity to the floor of the right orbit.	Enucleation with curettage, five years follow-up data with no recurrence.
Sheethal et al. (2019) [12]	15 years/female	Pain, swelling, and pus discharge with respect to the left 26 and 27 regions for three months. Missing 28.	Ill-defined, radiolucent lesion associated with an impacted third molar displaced to the left maxillary sinus.	Single large destructive lesion involving lateral and posterior wall of the maxillary sinus.	Enucleation along with tooth number 28 removal. No follow-up data.
Goto et al. (2020) [13]	21 years/male	No clinical symptoms or any extraoral or intra-oral swelling associated with the lesion.	Upper right third molar which was displaced into the maxillary sinus.	A dense cystic lesion that entirely occupied the right maxillary sinus extending to the right pterygoid process of the sphenoid bone near the skull base.	Enucleation without the use of Carnoy’s solution 20 months after the initial surgery.
Walsh et al. (2022) [14]	29 years/female	Right side retro-orbital pain for four months and loose upper right molar.	An ectopic tooth in the right maxillary sinus.	Cystic mass in right maxillary sinus and opacification of the maxillary and ethmoidal air cells.	Marsupialization post six months follow up. No recurrence.

The case reports of maxillary sinus OKC compiled in the table including ours were all non-syndromic. OKCs cover a wide age range, from the first decade of life to as late as the ninth decade [2,3]. The peak occurrence is seen in the second and third decades, which was similar to the reported case [3,6].

In the current case, the cyst extended into the floor of the orbit, hard plate, and pterygoid plates similar to the case reported by Goto et al. [13]. The majority of the maxillary sinus OKCs reported were symptomatic although this case was asymptomatic [2,4,7-9]. As per the literature, 25% to 40% of the cases have involvement of an unerupted impacted, displaced, or incompletely formed tooth [2,4,7-14]. In the reported case, there was no association with either any impacted or undeveloped tooth or any discharge associated with the lesion.

OKCs have a multifactorial theory of origin. Initially, the primordium of the tooth was thought by many authors to be the origin of these cysts and hence the name primordial cyst but now the dental lamina is considered to be the most likely origin. The basal cell layer of the oral epithelium is also thought to possibly play a role in the aetiology of these cysts [3]. The origin of OKC in the maxillary sinus is controversial, presumably arising from the entrapment of odontogenic epithelium within the sinus because of the close anatomy. A breach in the Schneiderian membrane due to odontogenic infection or odontogenic pathology of the maxillary bone can lead to maxillary sinus infection [14].

The theory of intrinsic growth potential of a cyst due to expression of Ki67, proliferating cell nuclear antigen (PCNA), and p-63 has also been proposed according to which patients who have the predisposition to form keratocyst will always have a higher risk of developing a cyst as long as a dental lamina or its remnant are present [15]. As per studies, Ki67 labelling is higher in cases with PTCH1 mutations. The theory specifically can be applied to syndromic patients (mutation rate > 85%) but sporadic OKC cases cannot be excluded (mutation rate < 30%). In the case of sporadic cases where there is epithelium separation from the connective tissue of the cyst, the PTCH1 mutation rate increases to 84% nearly equal to the syndromic OKC [16].

This theory could also be applied to the current case considering its behaviour and invasive potential but confirmation required evidence through genomic testing and immunohistochemistry (IHC) expression, which could not be done due to limited resources. Also, in the reported case, the cyst could have developed as a result of entrapment and proliferation of odontogenic epithelial cells or extensions of the basal cell layer of the epithelium of oral mucosa in the sinus. The patient had very poor oral hygiene and severe periodontitis, which could have additionally attributed to the infection of the sinus.

Several theories have been proposed for explaining the invasive and destructive nature of OKC. Growth in OKC is linked to unknown growth factors inherent in the epithelium itself or enzymatic activity in the fibrous wall [15]. Its invasion and infiltration are attributed to the multicentric growth potential that is cystic growth brought about by the proliferation of local groups of epithelial cells [17]. In the current case, the cyst was unilocular and there was no multicellularity seen in histopathology yet it was large, expansile, destroying the sinus floor, and perforating the cortical bone.

Aggressive maxillary sinus OKCs tend to penetrate into the surrounding soft tissues with expansion and perforation of cortical bone. The expansion has been reported to occur in up to 60% of cases, similar to the current case reported [3]. Advanced imaging techniques like CT and MRI are more useful as sometimes the pan tomographs can be misleading in viewing large lesions involving the maxillary sinus and those invading the skull base or surrounding spaces [13,18]. All case reports of sinus involvement used CT as their chief source of investigation as it not only demarcates the clear boundaries and extent of the lesion but also helps to demonstrate other features of OKCs, such as bony changes (expansion in buccolingual/palatal direction and erosion), internal density, and extension into soft tissue [19]. It also provides a better aid for the surgeons to prepare their procedures pre-operatively. Even in our case, OPG and the paranasal view did not give a clear interpretation of the lesion while CT demarcated the lesion boundaries discretely.

There is no universally accepted treatment for OKC. Considering its aggressive nature and history of recurrence, the primary aim of treatment is to achieve total eradication. The techniques involve decompression followed by enucleation and peripheral osteotomy, which show less recurrence, as compared to only enucleation, which has a recurrence rate of 17-56% [13]. The application of Carnoy’s solution has been very effective in adjunct to peripheral osteotomy and enucleation in extensive lesions in reducing the recurrence rate [9]. For our case too, freshly prepared Carnoy’s solution was used post-enucleation and curettage. Even with Carnoy’s solution application, a recurrence rate of 1-8.7% is reported and hence patient has been kept under periodic follow-up [13].

## Conclusions

Maxillary sinus OKCs are less in occurrence and every case has a varied presentation. FNAC is of limited help and CT imaging should be considered as the baseline radiological investigation to diagnose and plan treatment. Histopathology, genomic testing, and IHC should be considered as diagnostic criteria as they add better explanatory data on the aetiology and behaviour patterns of OKCs. Post-operative periodic follow-up should be mandatory irrespective of the operative procedure followed.
